# Supplementary material for: Causal Relationship between Aging and Anorexia Nervosa: A White-Matter-Microstructure-Mediated Mendelian Randomization Analysis
Source: Biomedicines. 2024 Aug 16;12(8):1874. doi: 10.3390/biomedicines12081874 (PMC11351342; doi:10.3390/biomedicines12081874)
Supplement: Supplementary file 1 [file biomedicines-12-01874-s001.zip › Supplementary Figure/Figure S4/Singe SNP plot of MR analysis for TL to ubm-b-1954.pdf]

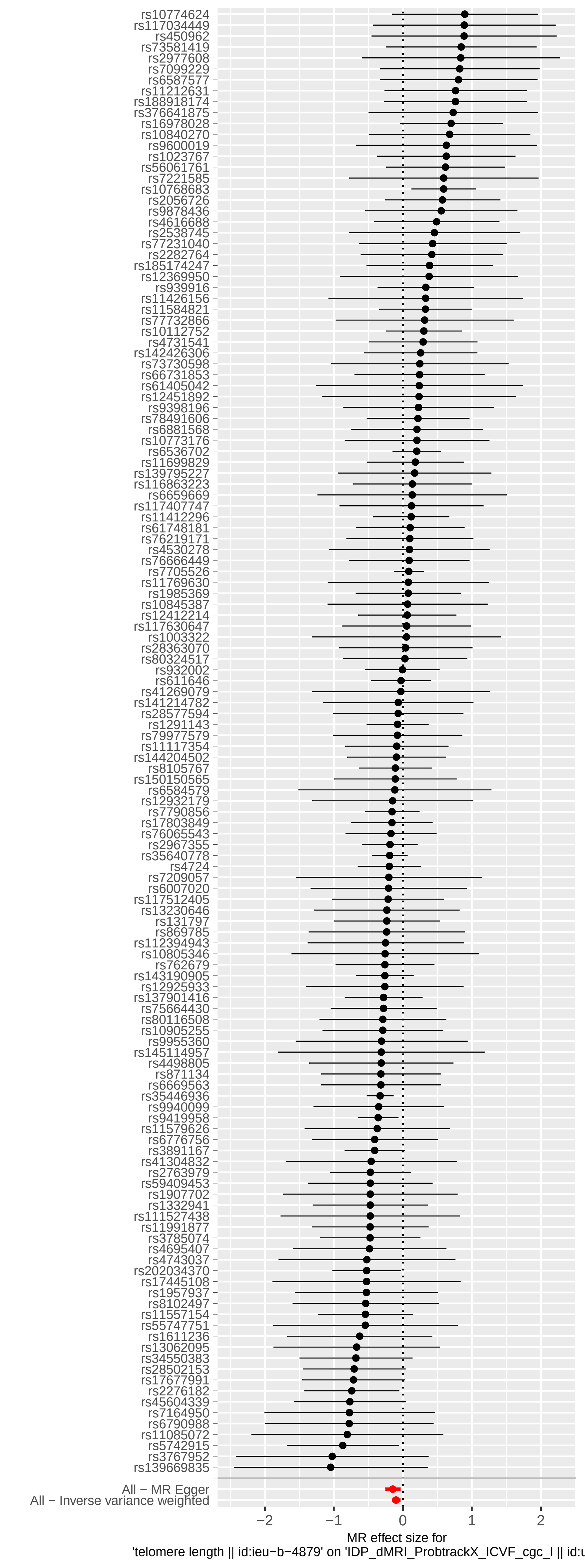

rs10774624
rs117034449
rs450962
rs73581419
rs2977608
rs7099229
rs6587577
rs11212631
rs188918174
rs376641875
rs16978028
rs10840270
rs9600019
rs1023767
rs56061761
rs7221585
rs10768683
rs2056726
rs9878436
rs4616688
rs2538745
rs77231040
rs2282764
rs185174247
rs12369950
rs939916
rs11426156
rs11584821
rs77732866
rs10112752
rs4731541
rs142426306
rs73730598
rs66731853
rs61405042
rs12451892
rs9398196
rs78491606
rs6881568
rs10773176
rs6536702
rs11699829
rs139795227
rs116863223
rs6659669
rs117407747
rs11412296
rs61748181
rs76219171
rs4530278
rs76666449
rs7705526
rs11769630
rs1985369
rs10845387
rs12412214
rs117630647
rs1003322
rs28363070
rs80324517
rs932002
rs611646
rs41269079
rs141214782
rs28577594
rs1291143
rs79977579
rs11117354
rs144204502
rs8105767
rs150150565
rs6584579
rs12932179
rs7790856
rs17803849
rs76065543
rs2967355
rs35640778
rs4724
rs7209057
rs6007020
rs117512405
rs13230646
rs131797
rs869785
rs112394943
rs10805346
rs762679
rs143190905
rs12925933
rs137901416
rs75664430
rs80116508
rs10905255
rs9955360
rs145114957
rs4498805
rs871134
rs6669563
rs35446936
rs9940099
rs9419958
rs11579626
rs6776756
rs3891167
rs41304832
rs2763979
rs59409453
rs1907702
rs1332941
rs111527438
rs11991877
rs3785074
rs4695407
rs4743037
rs202034370
rs17445108
rs1957937
rs8102497
rs11557154
rs55747751
rs1611236
rs13062095
rs34550383
rs28502153
rs17677991
rs2276182
rs45604339
rs7164950
rs6790988
rs11085072
rs5742915
rs3767952
rs139669835
All − MR Egger
All − Inverse variance weighted
−2
−1
0
1
2
MR effect size for
'telomere length || id:ieu−b−4879' on 'IDP_dMRI_ProbtrackX_ICVF_cgc_l || id:u
